# Hydrogen Production Dynamics During Lactulose Breath Testing in Patients with Suspected SIBO

**DOI:** 10.3390/jcm15114189

**Published:** 2026-05-28

**Authors:** Monika Waśkow, Magdalena Tańska, Sebastian Glowinski

**Affiliations:** 1 Institute of Health Sciences, Pomeranian University of Slupsk, 76-200 Slupsk, Poland; monika.waskow@upsl.edu.pl (M.W.); magdalena.tanska@upsl.edu.pl (M.T.); 2 Institute of Physical Culture, The State Academy of Applied Sciences in Koszalin, 75-582 Koszalin, Poland

**Keywords:** small intestinal bacterial overgrowth (SIBO), lactulose breath test, hydrogen production, intestinal fermentation, breath test dynamics

## Abstract

**Background:** Small intestinal bacterial overgrowth (SIBO) is characterized by excessive microbial colonization of the small intestine and is commonly diagnosed using hydrogen breath tests. However, most studies focus primarily on diagnostic thresholds rather than the overall dynamics of hydrogen production during the test. **Methods:** This cross-sectional study included 162 adults with chronic gastrointestinal symptoms who underwent a lactulose hydrogen breath test. Hydrogen concentrations were measured every 20 min over a 180 min period. Total hydrogen production was quantified using the area under the concentration–time curve (AUC), and hydrogen levels during the early (0–60 min) and late (>60 min) phases of the test were analyzed. **Results:** Among the participants, 100 (61.7%) were classified as SIBO-positive and 62 (38.3%) as SIBO-negative. Individuals with SIBO exhibited significantly higher total hydrogen production compared with those with a negative breath test result (mean AUC: 6938 vs. 2292 ppm × min, *p* < 0.001). Early hydrogen levels were also higher in the SIBO-positive group (8.7 vs. 4.5 ppm, *p* < 0.001). The most pronounced difference was observed during the late phase of the test, where hydrogen concentrations were markedly elevated in SIBO-positive individuals (52.7 vs. 12.0 ppm, *p* < 0.001). **Conclusions:** SIBO is associated with markedly increased hydrogen production during lactulose breath testing, particularly during the later stages of the test. These findings improve understanding of hydrogen production dynamics during lactulose breath testing and provide additional descriptive information regarding hydrogen response patterns; however, further studies are needed before clinical application. The observed differences should also be interpreted within the applied breath test classification framework.

## 1. Introduction

Small intestinal bacterial overgrowth (SIBO) is a condition characterized by excessive colonization of the small intestine by bacteria typically found in the colon [1,2,3,4]. This phenomenon may lead to increased fermentation of carbohydrates within the small intestine, resulting in the production of intestinal gases, primarily hydrogen and methane [5]. Consequently, patients may develop a variety of gastrointestinal symptoms, including abdominal bloating, abdominal pain, diarrhea, constipation, and a sensation of excessive intestinal gas or abdominal rumbling [6].

Hydrogen breath tests are among the most commonly used diagnostic tools for SIBO, as they provide a non-invasive method for assessing intestinal gas production resulting from bacterial fermentation [7,8]. One of the most frequently applied diagnostic procedures is the lactulose hydrogen breath test. During the test, hydrogen concentrations in exhaled air are measured at defined time intervals following the administration of a fermentable substrate [9]. Lactulose was selected as the test substrate because it enables assessment of hydrogen production dynamics throughout the entire duration of intestinal transit, including both early and late fermentation phases, and is widely used in clinical practice for hydrogen breath testing. An increase in hydrogen concentration above a predefined threshold within a specified time after lactulose ingestion is interpreted as a positive result suggestive of SIBO [1,7].

The interpretation of lactulose breath test results may be influenced by several gastrointestinal factors, including variations in orocecal transit time, intestinal motility disorders, and underlying gut microbiota composition. In addition, conditions such as irritable bowel syndrome and altered intestinal permeability may affect hydrogen production and contribute to variability in test outcomes [10,11,12]. These factors may complicate the interpretation of breath test results and highlight the need for more comprehensive approaches to analyzing hydrogen production dynamics.

In most previous studies, the interpretation of breath test results has focused primarily on simple diagnostic criteria, such as the maximum increase in hydrogen concentration or the difference between baseline and peak hydrogen levels within a specified time interval [7,13,14]. In contrast, the complete temporal profile of hydrogen concentration changes and the overall hydrogen production throughout the duration of the test have been evaluated less frequently [15,16]. The present study focuses primarily on the temporal dynamics and cumulative characteristics of hydrogen production during breath testing rather than exclusively on conventional threshold-based interpretation.

However, analysis of hydrogen production dynamics during breath testing may provide additional insight into both the intensity and timing of intestinal fermentation processes [17]. Parameters such as the area under the hydrogen concentration–time curve (AUC) or differences between early and late phases of gas production may reflect altered fermentation patterns in patients with SIBO [18].

While our previous analysis of this cohort examined potential associations between SIBO and systemic laboratory parameters, the current study focuses on the temporal dynamics of hydrogen production during lactulose breath testing. Most previous studies have primarily relied on threshold-based interpretations of breath test results, such as peak hydrogen levels or predefined diagnostic cut-offs, which provide limited insight into the overall pattern and intensity of intestinal fermentation. In contrast, the temporal profile of hydrogen production remains relatively underexplored.

Therefore, the primary aim of this exploratory study was to evaluate temporal patterns of hydrogen production during the lactulose hydrogen breath test in patients presenting with gastrointestinal symptoms, with particular emphasis on total hydrogen production and differences between the early and late phases of fermentation in individuals with positive and negative lactulose hydrogen breath test results suggestive of SIBO.

We hypothesized that hydrogen production during lactulose breath testing differs between SIBO-positive and SIBO-negative individuals and that analysis of these dynamics may provide additional descriptive insight beyond conventional threshold-based assessment, without implying independent diagnostic value. Because group classification was based on established hydrogen breath test criteria, the present analysis should be interpreted as exploratory characterization of hydrogen response patterns within the applied diagnostic framework. The present analysis was exploratory and was not designed to establish new diagnostic criteria. A more detailed characterization of hydrogen production dynamics may have implications for the clinical interpretation of breath test results.

## 2. Materials and Methods

### 2.1. Participants

The study had a cross-sectional design and was carried out at the Interdisciplinary Center for Research on Civilizational Diseases, Pomeranian University in Słupsk, Poland. In total, 162 adults experiencing gastrointestinal symptoms for a period of at least three months were enrolled. The dataset used in this analysis partially overlaps with a previously published study evaluating systemic laboratory parameters in the same study population [4]; however, the present analysis focuses on hydrogen production dynamics during breath testing.

The study was conducted in accordance with the principles outlined in the Declaration of Helsinki. Written informed consent was obtained from all participants prior to their inclusion in the study. The study protocol received approval from the Bioethics Committee of the Medical Chambers in Gdańsk, Poland (Approval No. KB-15/23).

Eligibility criteria included age of 18 years or older, the presence of gastrointestinal symptoms lasting at least three months, and provision of written informed consent. Individuals were excluded if they were younger than 18 years, pregnant, had undergone abdominal surgery within the previous six months, had a colonoscopy or fluoroscopy within the last four weeks, received antibiotic therapy within four weeks before testing, used probiotics or prebiotics within two weeks prior to the test, smoked within 12 h before the HBT, or did not provide informed consent.

### 2.2. Hydrogen Breath Test Protocol

Before undergoing the hydrogen breath test (HBT), participants completed a structured questionnaire to confirm eligibility criteria and collect basic clinical and sociodemographic information. Participants also received detailed instructions regarding test preparation. They were asked to discontinue the use of vitamin preparations and dietary supplements at least three days before the examination. On the day preceding the test, participants followed a restricted pre-test diet intended to minimize intestinal fermentation and reduce baseline hydrogen production before breath testing. Foods containing fermentable carbohydrates were avoided, including bread, potatoes, lactose-containing dairy products, fruit juices, high-fiber foods, and products known to increase intestinal gas production, such as onions, garlic, cabbage, legumes, and fermented vegetables. Although some of the dietary restrictions overlapped with low-FODMAP principles, the preparation protocol was not intended as a formal low-FODMAP dietary intervention but rather reflected standard pre-test recommendations used before hydrogen breath testing.

Participants were required to fast for at least 14 h before the examination. They were also instructed to refrain from smoking and chewing gum for a minimum of 12 h prior to testing. During the fasting period, small amounts of plain water were permitted to prevent dehydration before anthropometric and body composition assessments.

Anthropometric measurements were obtained using standardized procedures. Body weight and height were measured with calibrated equipment, and body mass index (BMI) was calculated as body weight in kilograms divided by the square of height in meters (kg/m^2^). Waist and hip circumferences were measured according to a standardized anthropometric protocol.

Body composition was evaluated using bioelectrical impedance analysis (BIA) with the TANITA SC-240 MA body composition analyzer (Tanita Corporation, Tokyo, Japan). The analysis provided estimates of several parameters, including body fat percentage, muscle mass, bone mass, total body water percentage, visceral fat rating, metabolic age, and basal metabolic rate (BMR).

To confirm abstinence from smoking prior to the examination, exhaled carbon monoxide (CO) was measured using a Smokerlyzer Micro+ device (Bedfont Scientific Ltd., Kent, UK). Participants with CO concentrations exceeding 6 ppm were excluded from further analysis.

**Figure 1:**
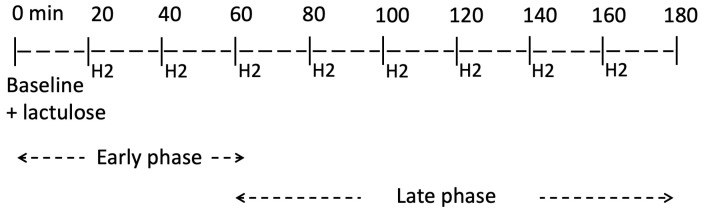
Schematic overview of the lactulose hydrogen breath test protocol. Hydrogen concentrations were measured every 20 min for 180 min following lactulose ingestion. The early phase was defined as 0–60 min and the late phase as >60 min.

The lactulose hydrogen breath test was performed using the Gastrolyzer Gastro+ device (Bedfont Scientific Ltd., Harrietsham, UK). The analyzer measures hydrogen levels in exhaled breath; methane concentrations were not assessed, and intestinal methanogen overgrowth (IMO) was therefore not evaluated. After collection of the baseline fasting breath sample, participants ingested 10 g of lactulose (15 mL of lactulose syrup) diluted in 200 mL of water. Subsequent breath samples were collected every 20 min over a total testing period of 180 min (Figure 1).

Hydrogen concentrations were recorded in parts per million (ppm) at each measurement point. To further characterize hydrogen production during the test, additional parameters were calculated. Total hydrogen production was expressed as the area under the hydrogen concentration curve (AUC) across the entire testing period. Moreover, hydrogen production was analyzed in two-time intervals: an early phase (0–60 min) and a late phase (>60 min).

Participants were classified as breath test-positive or breath test-negative according to established hydrogen breath test criteria suggestive of SIBO, defined as an increase in exhaled hydrogen concentration of at least 20 ppm above baseline within 90 min after lactulose ingestion. Dynamic parameters such as AUC and phase-specific hydrogen levels were not used for group classification and were calculated post hoc solely for descriptive analysis.

### 2.3. Statistical Analysis

All 162 participants were included in the statistical analysis. Prior to analysis, the dataset was checked for completeness, internal consistency, and plausibility. All statistical analyses were conducted using the R statistical environment (R version 4. 5. 2 Foundation for Statistical Computing, Vienna, Austria). Data processing and graphical visualization were performed using commonly applied R packages, and figures were generated with the ggplot2 package.

The distribution of continuous variables was evaluated using the Shapiro–Wilk test. Homogeneity of variances between groups was assessed using Levene’s test with the Brown–Forsythe correction. Continuous variables are presented as mean values with standard deviations (SD) or, where appropriate, as mean values with corresponding 95% confidence intervals (95% CI).

Comparisons between participants with positive and negative hydrogen breath test results (SIBO-positive vs. SIBO-negative) were performed using the independent samples Student’s *t*-test when the assumption of equal variances was met. In cases where variances were unequal, Welch’s *t*-test was applied. If the assumption of normality was not satisfied, the Mann–Whitney U test was used as a non-parametric alternative.

Hydrogen production during the breath test was characterized using several derived parameters. Total hydrogen production was quantified using the area under the hydrogen concentration–time curve (AUC), calculated using the trapezoidal rule based on measurements obtained at each time point. Baseline values were included in the calculation. Hydrogen concentrations were expressed in parts per million (ppm), and AUC values are reported as ppm × min. All calculations were performed using the R statistical environment. In addition, hydrogen production was analyzed in two predefined time intervals: the early phase (0–60 min) and the late phase (>60 min).

To investigate whether selected demographic and anthropometric characteristics were independently associated with SIBO status, a multivariable logistic regression model was constructed. The model included age, sex, body mass index (BMI), and visceral fat level as potential predictors. Regression results are presented as estimated coefficients together with their standard errors and corresponding *p*-values.

All statistical tests were two-sided, and a *p*-value below 0.05 was considered statistically significant. In addition, effect size was calculated using Cohen’s d to quantify the magnitude of differences in hydrogen production between SIBO-positive and SIBO-negative groups. No formal sample size calculation was performed, as the study was based on an available clinical cohort and should be considered exploratory.

## 3. Results

### 3.1. Study Population Characteristics

**Figure 2:**
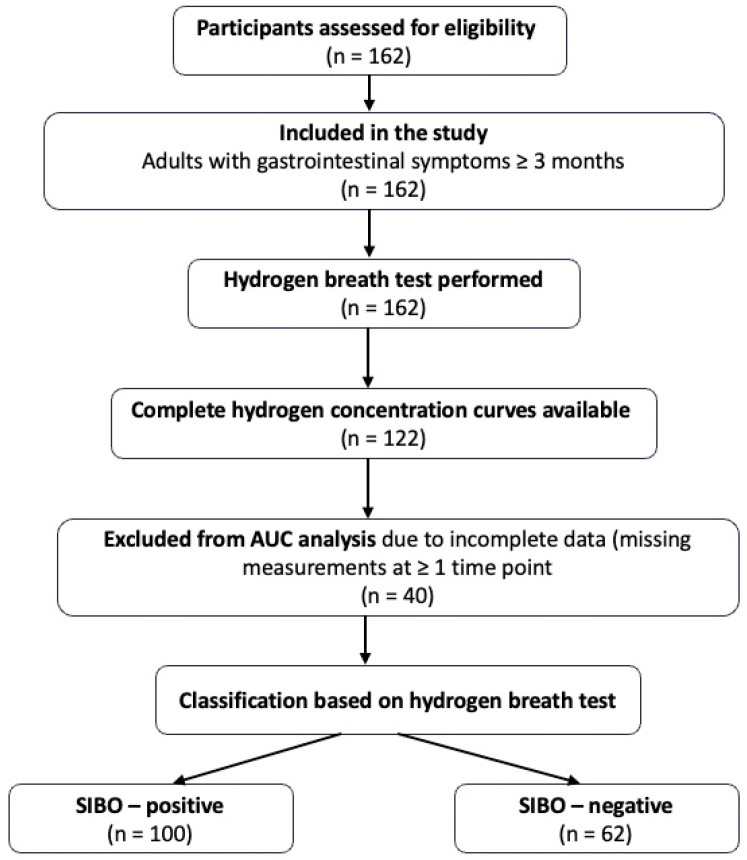
Flow diagram of participant selection and inclusion in the analysis.

The study included 162 participants who underwent a lactulose hydrogen breath test (Figure 2). Based on established hydrogen breath test criteria suggestive of SIBO, 100 individuals (61.7%) were classified as breath test-positive and 62 (38.3%) as breath test-negative.

The mean age of the study population was 44.4 ± 12.6 years. Participants in the SIBO-negative group were slightly older than those in the SIBO-positive group (47.1 ± 13.3 vs. 42.7 ± 11.8 years, *p* = 0.032). However, this difference did not remain statistically significant after adjustment for potential confounders in the multivariable logistic regression model (Table 1). No statistically significant differences were observed between the groups in anthropometric parameters. No significant differences in AUC were observed between male and female participants (*p* = 0.37), indicating that hydrogen production dynamics were not influenced by sex. Body mass index (BMI) was comparable between SIBO-positive and SIBO-negative individuals (25.05 vs. 25.43 kg/m^2^, *p* = 0.607). Similarly, visceral fat level did not differ significantly between groups, although slightly higher values were observed in the SIBO-negative group (7.3 vs. 6.2, *p* = 0.064). A detailed comparison of baseline characteristics according to SIBO status is presented in Table 1.

### 3.2. Multivariable Logistic Regression Analysis

To evaluate whether selected demographic and anthropometric variables were independently associated with SIBO status, a multivariable logistic regression model was constructed including age, sex, body mass index (BMI), and visceral fat level as potential predictors. The results of the regression analysis are presented in Table 2. None of the analyzed variables showed a statistically significant independent association with SIBO status after adjustment for the other covariates included in the model. Age demonstrated a negative regression coefficient, suggesting a tendency toward a lower probability of SIBO with increasing age; however, this association was not statistically significant (*p* = 0.418). Sex was also not associated with SIBO status in the adjusted model (*p* = 0.930). Anthropometric parameters likewise did not show significant associations with SIBO. Body mass index (BMI) showed a small positive regression coefficient but did not reach statistical significance (*p* = 0.614). Visceral fat level demonstrated a negative coefficient, indicating a tendency toward lower SIBO prevalence with higher visceral adiposity, although this relationship was not statistically significant (*p* = 0.562). Overall, the multivariable analysis indicates that age, sex, BMI, and visceral fat level were not independent predictors of SIBO in the analyzed population.

### 3.3. Hydrogen Production During Lactulose Breath Testing

Hydrogen breath test data were available for all participants included in the analysis (n = 162). In cases where measurements at one or more time points were missing, the area under the curve (AUC) was calculated using the trapezoidal rule based on available data points without interpolation or imputation. This approach allowed inclusion of all participants while preserving the integrity of the observed hydrogen profiles.

Total hydrogen production was quantified using the area under the hydrogen concentration–time curve (AUC). The results of the comparison between SIBO-positive and SIBO-negative individuals are presented in Table 3. Mean hydrogen AUC was substantially higher in participants diagnosed with SIBO compared with individuals with a negative breath test result. The magnitude of this difference corresponded to a large effect size (Cohen’s d = 1.35, 95% CI: 1.11–1.59), indicating a substantial difference in hydrogen production between groups beyond statistical significance.

**Figure 3:**
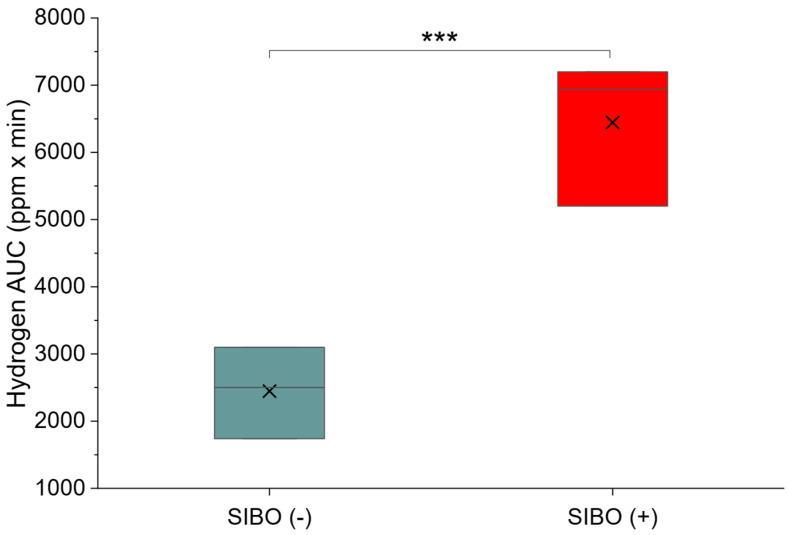
Comparison of hydrogen AUC during the lactulose breath test between SIBO-positive and SIBO-negative individuals. Boxplots show the median, interquartile range (IQR), whiskers, and outliers. *** *p* < 0.001.

Specifically, the mean AUC value was 6938 ppm × min in the SIBO-positive group, whereas in SIBO-negative participants the mean AUC was 2292 ppm × min (Figure 3). This corresponds to approximately a threefold higher total hydrogen production during the breath test in individuals with SIBO. The difference between groups was highly statistically significant (*p* = 5.0 × 10^−15^). These results indicate markedly increased hydrogen production during lactulose fermentation in individuals with SIBO.

### 3.4. Early Hydrogen Production

Hydrogen production during the early phase of the lactulose breath test (0–60 min) was evaluated to compare initial hydrogen concentrations between groups. Mean early hydrogen levels were significantly higher in individuals with SIBO than in participants with a negative breath test result. Specifically, the mean early hydrogen concentration was 8.7 ppm in the SIBO-positive group and 4.5 ppm in SIBO-negative individuals. The difference between groups was statistically significant (*p* = 3.2 × 10^−6^). Although hydrogen levels were higher in the SIBO-positive group during the early phase of the test, the magnitude of the difference between groups was smaller than that observed during the late phase of the test.

### 3.5. Late Hydrogen Production

**Figure 4:**
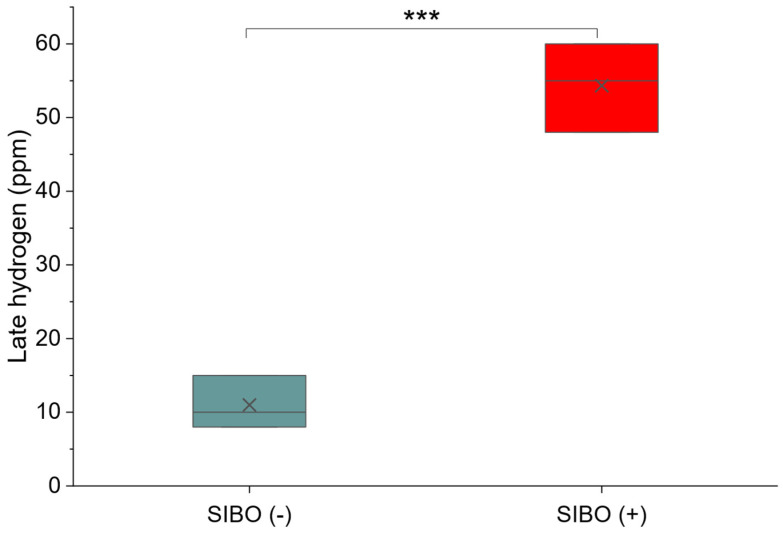
Comparison of late hydrogen production during the lactulose breath test between SIBO-positive and SIBO-negative individuals. Boxplots show the median, interquartile range (IQR), whiskers, mean values, and outliers. *** *p* < 0.001.

Hydrogen production during the late phase of the lactulose breath test (>60 min) differed markedly between the analyzed groups. Mean late hydrogen concentrations were substantially higher in individuals with SIBO than in participants with a negative breath test result (Figure 4). Specifically, the mean late hydrogen level was 52.7 ppm in the SIBO-positive group, whereas in SIBO-negative individuals the mean value was 12.0 ppm. This represents more than a fourfold difference in hydrogen concentration during the late phase of the test. The difference between groups was highly statistically significant (*p* < 2.2 × 10^−16^), as shown in Table 3. These findings demonstrate markedly increased hydrogen production during the later stages of the breath test in individuals with SIBO.

### 3.6. Association Between Late Hydrogen Levels and Total Hydrogen Production

**Figure 5:**
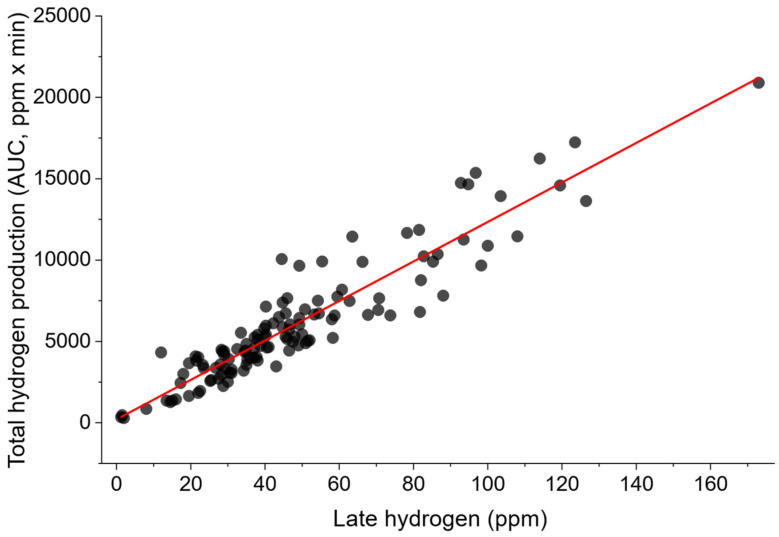
Association between late hydrogen concentrations and total hydrogen production (AUC) during the lactulose hydrogen breath test. Each point represents one participant. The red line indicates the linear regression trend. Higher late-phase hydrogen concentrations were associated with greater cumulative hydrogen production.

A strong positive association was observed between hydrogen concentrations measured during the late phase of the lactulose breath test and total hydrogen production expressed as the area under the concentration–time curve (AUC). As shown in Figure 5, individuals with higher late hydrogen levels consistently exhibited greater cumulative hydrogen production. Spearman correlation analysis demonstrated a very strong relationship between these parameters (ρ = 0.98, *p* < 0.001). Linear regression analysis further showed that late hydrogen levels explained a substantial proportion of the variability in total hydrogen production (R^2^ = 0.87).

### 3.7. Temporal Patterns of Hydrogen Production

**Figure 6:**
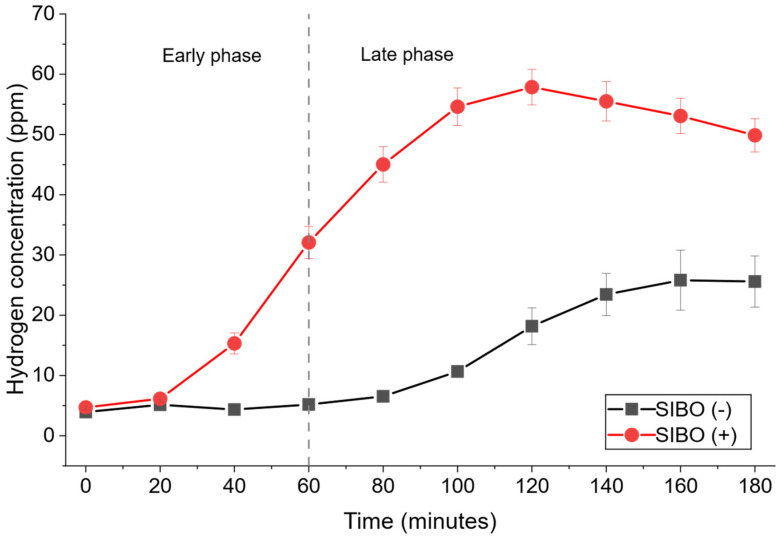
Temporal patterns of hydrogen production during the lactulose breath test in SIBO-positive and SIBO-negative individuals. The dashed vertical line indicates the transition from the early phase to the late phase of the test. Data are presented as mean ± SE.

Analysis of hydrogen production across the entire duration of the lactulose breath test revealed clear temporal differences between SIBO-positive and SIBO-negative individuals. The mean hydrogen concentration curves for both groups are presented in Figure 6. Participants with SIBO demonstrated consistently higher hydrogen concentrations throughout the breath test compared with individuals with a negative test result. In the SIBO-negative group, hydrogen levels remained relatively low and stable during most of the test period. In contrast, SIBO-positive individuals exhibited a progressive increase in hydrogen concentrations over time. The difference between groups became particularly pronounced during the later time points of the test, corresponding to the late phase of the breath test. During this period, hydrogen concentrations in SIBO-positive participants increased markedly, whereas levels in the SIBO-negative group remained substantially lower.

## 4. Discussion

The present study evaluated hydrogen production during lactulose breath testing in individuals with suspected small intestinal bacterial overgrowth. Participants classified as SIBO-positive showed substantially higher hydrogen production than SIBO-negative individuals, both in terms of total hydrogen production (AUC) and temporal changes observed during the test [1]. While breath test interpretation is usually based on predefined diagnostic thresholds, the present analysis focused on breath test responses throughout the entire test period.

One of the main findings was the markedly higher total hydrogen production observed in SIBO-positive participants. Throughout the manuscript, the terms SIBO-positive and SIBO-negative refer to classification based on lactulose hydrogen breath test criteria. The approximately threefold increase in AUC suggests greater microbial fermentation during intestinal transit of lactulose [19]. Because AUC reflects hydrogen production over the full duration of the test, it may better represent overall fermentation activity than single peak measurements. Lactulose is not absorbed in the small intestine and therefore remains available for bacterial fermentation throughout the gastrointestinal tract [16]. Hydrogen detected during breath testing is produced exclusively by intestinal microorganisms during carbohydrate metabolism [5,7], which may explain the higher hydrogen levels observed in individuals with suspected bacterial overgrowth.

The most pronounced differences between groups were observed during the late phase of the breath test. This may reflect prolonged or more intense fermentation in SIBO-positive individuals [20]. However, late hydrogen peaks should be interpreted with caution. Under physiological conditions, lactulose eventually reaches the colon, where substantial bacterial fermentation also takes place [17]. Therefore, increased hydrogen concentrations during the later stages of the test may partly reflect colonic fermentation rather than exclusively small intestinal bacterial activity, particularly in individuals with rapid orocecal transit [8,13]. Because orocecal transit time was not assessed directly in the present study, the exact site of hydrogen production could not be determined.

The observed temporal pattern, with both elevated early hydrogen levels and a marked increase during the late phase of the test, likely reflects the combined effects of small intestinal and colonic fermentation. Hydrogen breath test results may be influenced by several factors, including intestinal motility, gut microbiota composition, and the presence of methane-producing microorganisms. In addition, gastrointestinal conditions such as irritable bowel syndrome and altered intestinal permeability may affect hydrogen production dynamics, although these factors were not assessed in the present study.

The present findings may also have implications for the interpretation of lactulose breath tests in clinical practice. Current diagnostic approaches are based mainly on predefined hydrogen thresholds [1], whereas parameters such as AUC and phase-specific hydrogen production may provide additional information about fermentation dynamics. This may be particularly relevant in patients with borderline or inconclusive breath test results. However, hydrogen breath testing remains methodologically challenging, and the optimal diagnostic thresholds and clinical interpretation of test results are still debated [7,9].

Group classification was based on established hydrogen breath test criteria. Therefore, the observed differences in hydrogen production should be interpreted with caution. Since SIBO status was determined using hydrogen response patterns during the breath test, parameters such as AUC and phase-specific hydrogen levels are not independent of the diagnostic definition itself. Therefore, these findings should be interpreted as descriptive observations rather than independent diagnostic markers [21]. Accordingly, the observed differences partly reflect the applied classification framework and should not be interpreted as evidence of independent diagnostic performance.

Interestingly, demographic and anthropometric variables were not significantly associated with SIBO status in the multivariable analysis. Age, sex, BMI, and visceral fat level did not appear to substantially influence breath test responses in the analyzed cohort. However, other factors, including intestinal microbiota composition and gastrointestinal motility, may still contribute to the observed variability.

Despite these limitations, the present study provides additional insight into hydrogen production patterns during lactulose breath testing. Chronic bacterial overgrowth may also contribute to metabolic disturbances, including vitamin B12 deficiency related to bacterial cobalamin utilization [22]. Future studies combining breath test dynamics with microbiome analysis, methane measurements, and longitudinal clinical follow-up may help to better understand the mechanisms and clinical significance of the observed hydrogen production dynamics.

In summary, individuals with SIBO demonstrated increased hydrogen production and distinct temporal patterns during lactulose breath testing. Analysis of hydrogen production dynamics may provide additional information beyond conventional threshold-based interpretation; however, further studies are needed to determine the clinical relevance of these findings. The observed differences should be interpreted within the applied classification framework and do not imply independent diagnostic significance.

## 5. Limitations and Future Directions

Several limitations of this study should be acknowledged. First, group classification was based on hydrogen breath test criteria, and therefore some of the observed differences in hydrogen dynamics may be related to the diagnostic definition itself. Although jejunal aspirate culture is generally regarded as the reference method for SIBO diagnosis, lactulose hydrogen breath testing remains widely used in clinical practice because it is non-invasive and easier to perform. Nevertheless, no direct microbiological assessment, such as jejunal aspirate culture, was performed in the present study, which limits the ability to confirm SIBO using a reference method [23]. Second, the cross-sectional design does not allow conclusions about causal relationships between fermentation profiles and gastrointestinal symptoms. In addition, no formal sample size or power calculation was performed because the study was based on an available clinical cohort. Longitudinal studies are needed to determine whether the observed hydrogen responses change over time or in response to treatment. Another limitation is that lactulose breath test results may be influenced by variations in orocecal transit time, which can affect the timing of hydrogen production and contribute to variability in breath test results. Finally, phase angle, a potentially informative parameter derived from bioimpedance analysis, was not available in the present dataset and therefore could not be included in the analysis.

The study also relied on hydrogen breath testing as an indirect measure of intestinal bacterial fermentation. Although this method is widely used in clinical practice, breath test results may be affected by several physiological factors, including intestinal transit time, substrate delivery to the colon, and differences in gut microbiota composition between individuals. Methane concentrations were not measured in the present study. This is important because methanogenic microorganisms consume hydrogen and produce methane, which may result in lower measured hydrogen levels despite active intestinal fermentation. As a result, some individuals may have been misclassified based on hydrogen measurements alone. In addition, the lack of methane assessment prevented the identification of individuals with intestinal methanogen overgrowth, which may further influence hydrogen production patterns. These limitations highlight the importance of combined hydrogen–methane testing when interpreting breath test results.

Receiver operating characteristic (ROC) analyses were not performed because the aim of the present study was not to establish new diagnostic markers or validate diagnostic cut-off values. Instead, the analysis focused on describing hydrogen production dynamics in groups classified according to established SIBO criteria.

Another limitation is that the analysis focused mainly on breath test parameters and selected demographic and anthropometric variables. In addition, the study population included only individuals with gastrointestinal symptoms, which may limit the generalizability of the findings. Several potentially relevant confounding factors, including dietary variability before testing, gut microbiota composition, IBS subtypes, and concomitant medication use, were not fully controlled for and may have influenced hydrogen production during the test. Other factors associated with SIBO, such as gastrointestinal motility disorders, anatomical abnormalities, food intolerances, and detailed microbiome characteristics, were also not comprehensively evaluated.

An additional limitation is the lack of analysis of symptom profiles in relation to hydrogen production dynamics, which limits the direct clinical interpretation of AUC-related findings. Intestinal permeability was also not assessed, although lactulose is commonly used in permeability testing and altered epithelial barrier function may affect substrate availability and fermentation dynamics.

Future studies should further investigate the relationship between breath test dynamics and gut microbiota composition using molecular and sequencing-based approaches. Combining breath test parameters with microbiome analyses and clinical symptom assessment may help to better understand the mechanisms underlying altered fermentation patterns in individuals with suspected SIBO. Future research should also include combined hydrogen–methane testing to provide a more comprehensive evaluation of intestinal gas production and microbial activity. In addition, longitudinal and interventional studies may help determine whether changes in hydrogen production dynamics are associated with treatment response and clinical outcomes. Patient-centered outcomes should also be considered, as improvements in quality of life may not directly correlate with normalization of intestinal gas production [24].

## 6. Conclusions

The present study showed that individuals with small intestinal bacterial overgrowth had higher hydrogen production during the lactulose breath test, particularly during the later stages of the examination. SIBO-positive participants also demonstrated distinct temporal patterns of hydrogen production compared with individuals with negative breath test results. Analysis of hydrogen production dynamics may provide additional descriptive characterization of hydrogen response patterns during lactulose breath testing. However, further studies are needed to determine the clinical significance and potential practical application of these findings.

## Figures and Tables

**Table 1:** Baseline characteristics of the study population according to SIBO status.

Variables	SIBO (-) (n = 62)	Positive (n = 100)	*p*-Value
Age (years), mean ± SD	47.1 ± 13.3	42.7 ± 11.8	0.032 ^1^
BMI (kg/m^2^) mean ± SD	25.4 ± 4.6	25.1 ± 4.4	0.607 ^1^
Visceral fat level mean ± SD	7.3 ± 2.1	6.2 ± 1.9	0.064 ^1^
Sex (female/male), n (%)			0.794 ^2^
Female	49 (79.0)	82 (82.0)	
Male	13 (21.0)	18 (18.0)	
Age groups, n (%)			0.265 ^2^
<40 years	24 (38.7)	40 (40.0)	
40–60 years	28 (45.2)	52 (52.0)	
>60 years	10 (16.1)	8 (8.0)	

Abbreviations: SD—standard deviation; ^1^—Welch’s two-sample *t*-test. ^2^—Pearson’s Chi-squared test with Yates’ continuity correction.

**Table 2:** Multivariable logistic regression analysis for predictors of SIBO. Estimates are regression coefficients from multivariable logistic regression models.

Variables	β (Estimate)	SE	OR	*p*-Value
Age (years)	−0.021	0.026	0.98	0.418 ^1^
Sex	−0.054	0.622	0.95	0.930 ^1^
BMI	0.050	0.099	1.05	0.614 ^1^
Visceral fat level	−0.098	0.170	0.91	0.562 ^1^

Abbreviations: ^1^—Wald tests; SE—Standard error; OR—odds ratio (exp(β)).

**Table 3:** Hydrogen production parameters according to SIBO status.

Parameter	SIBO (-)	SIBO (+)	*p*-Value
Early hydrogen (ppm), mean ± SD	4.5 ± 3.1	8.7 ± 7.7	<0.001 ^1^
Hydrogen AUC (ppm × min), mean ± SD	2292 ± 1501	6938 ± 3632	<0.001 ^1^
Late hydrogen (ppm), mean ± SD	12.0 ± 10	52.7 ± 27	<0.001 ^1^
Body Mass Index (kg/m^2^), mean ± SD	25.4 ± 4.6	25.1 ± 4.4	0.677 ^2^

Abbreviations: SD—standard deviation; ^1^—Welch’s two-sample *t*-test, ^2^—UMW test.

## Data Availability

The data presented in this study are available on request from the corresponding author.
